# Evaluation of Lateral and Medial Parts of the Hamstring Muscle Fatigue Symmetry in Professional Footballers Cleared to Play After ACL Reconstruction

**DOI:** 10.3390/jcm13216521

**Published:** 2024-10-30

**Authors:** Łukasz Oleksy, Anna Mika, Martyna Sopa, Artur Stolarczyk, Olga Adamska, Miłosz Szczudło, Renata Kielnar, Magdalena Hagner-Derengowska, Rafał Buryta, Michał Jakub Nowak, Matylda Kowal, Jarosław Michał Deszczyński

**Affiliations:** 1 Department of Orthopedics, Traumatology and Hand Surgery, Faculty of Medicine, Wroclaw Medical University, 50-556 Wroclaw, Poland; loleksy@oleksy-fizjoterapia.pl; 2 Oleksy Medical & Sport Sciences, 37-100 Łańcut, Poland; 3 Institute of Clinical Rehabilitation, University of Physical Education in Kraków, 31-571 Kraków, Poland; 4 Institute of Applied Mechanics, Faculty of Mechanical Engineering, Poznan University of Technology, 61-138 Poznan, Poland; martyna.sopa@put.poznan.pl; 5 Department of Orthopaedics and Rehabilitation, Medical and Dentistry Faculty, Medical University of Warsaw, 02-091 Warsaw, Poland; artur.stolarczyk@wum.edu.pl (A.S.); jm.deszczynski@me.com (J.M.D.); 6 Department of Ophthalmology, Faculty of Medicine, Collegium Medicum Cardinal Stefan Wyszyński University in Warsaw, 01-815 Warsaw, Poland; o.adamska@uksw.edu.pl; 7 Centre of Sport and Recreation, University of Rzeszów, 35-959 Rzeszów, Poland; mszczudlo@ur.edu.pl; 8 Institute of Health Sciences, Medical College of Rzeszów University, 35-315 Rzeszów, Poland; kielnarrenata@o2.pl; 9 Laboratory of Geronto-prophylaxis, Centre for Innovative Research in Medical and Natural Sciences, Rzeszów University, 35-315 Rzeszów, Poland; 10 University Sports Center Nicolaus Copernicus University in Toruń, 87-100 Toruń, Poland; m.hagner-derengowska@umk.pl; 11 Institute of Physical Culture Sciences, University of Szczecin, 71-065 Szczecin, Poland; rafal.buryta@usz.edu.pl; 12 Faculty of Physical Culture Sciences, Collegium Medicum im. dr. Władysława Biegańskiego, Jan Długosz University in Częstochowa, 42-200 Częstochowa, Poland; michal.nowak@ujd.edu.pl; 13 Department of Physical Education, University of Rzeszow, 35-959 Rzeszów, Poland; mkowal@ur.edu.pl

**Keywords:** sEMG, hamstring muscle, symmetry, fatigue, ACL, football players, injury risk

## Abstract

**Objectives:** Rupture of the anterior cruciate ligament (ACL) is a severe injury common in sports. It also has a high rate of re-injury. The aim of this work was to assess hamstring muscle fatigue in active football players after ACL reconstruction who were cleared to play and to determine symmetry between the lateral and medial hamstring muscles. **Methods:** In professional football players post ACL reconstruction (n = 25) and non-injured players (n = 26), the bioelectrical activity of the medial (biceps femoris—BF) and lateral (semimembranosus and semitendinosus—SEM) hamstring muscles was measured during 60 s of isometric contraction. The fatigue variables were calculated using the Continuous Wavelet Transform (CWT) tool. **Results:** The football players following ACL reconstruction demonstrated significant asymmetry in fatigue of the lateral and medial hamstring muscles, with greater fatigue in the SEM compared to the BF muscle. Moreover, in those after reconstruction, the changes are more pronounced, with higher muscle fatigue in both limbs (they have lower MDF than non-injured players) and more severe SEM muscle insufficiency (noted in both limbs but with greater intensity in the non-operated one). **Conclusions:** The higher SEM muscle fatigue observed in this study influenced the lateral-to-medial activation ratio within the hamstring muscle, which may be a probable cause of this muscle’s insufficiency in laterally stabilizing the knee in the frontal and transverse plane. Furthermore, the hamstring muscles after reconstruction were more fatigued in both limbs, which may be another risk factor for ACL graft rupture. Therefore, increased fatigue in specific hamstring muscles may indicate the direction in which knee stabilization is compromised due to ACL overload. A muscle that becomes fatigued and inefficient more quickly also becomes ineffective in performing its function sooner, which can lead to increased overloading forces acting on the ACL graft.

## 1. Introduction

Rupture of the anterior cruciate ligament (ACL) is a severe injury that is common in sports [1,2]. Unfortunately, many athletes find it difficult to regain their previous performance levels after undergoing ACL reconstruction and face high rates of re-injury, including graft ruptures or injuries to the opposite leg [3,4,5]. The dynamic function of the hamstrings is essential for neuromuscular control of the knee during various daily activities [6,7]. In some studies, it has been examined how the quadriceps and hamstrings impacted ACL strain during knee movement and reported that hamstring contractions decrease tibia anterior translation and reduce ACL strain [2,6,8].

Evidence suggests that muscles crossing the knee adapt in varied ways after ACL injuries [2]. Individuals with ACL deficiencies show increased activity in the biceps femoris, medial gastrocnemius, and vastus lateralis muscles, while the semitendinosus muscle exhibits reduced activity [2]. Moreover, it has been noted that the medial and lateral hamstrings played distinct roles in controlling the knee joint in the coronal plane, which means that injuries to specific hamstring muscles can uniquely affect ACL strain [2,9]. The medial hamstrings contribute to the knee’s rotational, translational, and valgus stability [7]. It was reported that if the semitendinosus and gracilis tendons were harvested, it would lead to changes in the natural kinematics of the knee [7]. These findings indicate that medial hamstring insufficiency alters natural knee kinematics and stability, shifting the tibia to externally rotated and anteriorly translated positions [7].

Briem et al. [10] examined the activation patterns of medial versus lateral hamstrings in female athletes who had undergone ACL reconstruction with a hamstring graft. They exhibited inter-limb differences in medial versus lateral hamstring muscle activity not seen in uninjured controls, with the uninjured limb showing higher lateral hamstring activation [10]. Therefore, considering the distinct roles that the medial and lateral hamstrings play in knee kinematics, it becomes essential to conduct appropriate diagnostics that account for each part of the hamstring muscle. The biceps femoris exhibits greater activation compared to the medial hamstring muscles during high-velocity activities such as sprinting and jumping. This increased activation is essential for the generation of powerful knee flexion and hip extension, highlighting the functional differences among the hamstring muscle group [11]. Other researchers have evaluated the ratio between the lateral and medial parts of the hamstring by analyzing the amplitude of the EMG signal, indicating the level of engagement of each part during hamstring contraction [12,13]. They were able to determine which part, lateral or medial, worked harder in a given movement and which was less active [12,13]. Ratio analysis was conducted during walking and various exercises, and isometric contractions were examined in patients after ACL injuries and in those with knee osteoarthritis [12,13]. Rutherford et al. [12] assessed the medial-to-lateral hamstring ratio during treadmill walking in individuals with medial compartment knee osteoarthritis and indicated that it was altered, showing greater lateral hamstring activation in the symptomatic leg. Lynn et al. [13] also evaluated if the ratio of medial-lateral hamstring muscular activation could be altered with changes in foot rotation position during three standard lower-limb exercises.

In some studies, it has been reported that the slope of the EMG signal analyzed through wavelet transforms provided valuable insights into muscle fatigue by capturing time-frequency domain changes and complexity variations, making it a robust indicator for fatigue assessment [14]. Wavelet analysis is advantageous because it allows for the examination of non-stationary signals, such as EMG, in both time and frequency domains, which is crucial in accurately detecting fatigue [14,15]. It was reported that the changes in the frequency content of the EMG signal, such as shifts in the median or mean power frequencies, have been correlated with fatigue [14,15]. Therefore, the ratio between the medial and lateral parts of the hamstring, based on the measurement of fatigue during contraction assessed via EMG, may be a sensitive and useful indicator of hamstring muscle performance post ACL reconstruction.

Thus, the aim of this work was to assess hamstring muscle fatigue in active football players after ACL reconstruction who were cleared to play, as well as in non-injured players. The secondary objective was to determine whether this ratio differed between both knees in athletes after ACL reconstruction.

## 2. Materials and Methods

### 2.1. Participants

Male football players from professional teams were recruited for this study (Table 1) and divided into the following groups:

Group 1 (n = 25)—football players after ACL reconstruction (operated leg—after ACL reconstruction, non-operated leg—contralateral limb without ACL injury);

Group 2 (n = 26)—football players without injuries in the past three years (control group) (left limb equivalent of the operated limb and right limb equivalent of the non-operated limb).

The inclusion criteria for subjects following ACL reconstruction were as follows: regular football training; first and unilateral ACL rupture and reconstruction two–three years before the study; no additional injuries to the contralateral leg. All footballers were active players who were cleared to play. The control group included footballers who did not have any injuries recorded in the club’s medical documentation and did not self-report any injuries in the past three years. All participants performed a 10-minute warm-up before the tests.

All football players were informed about the research protocol and provided their written informed consent to participate in this study. Approval of the Ethical Committee at the Regional Medical Chamber in Kraków was obtained for the research (35/KBL/OIL/2024). All procedures were performed in accordance with the 1964 Declaration of Helsinki and its later amendments.

### 2.2. Procedures

#### 2.2.1. sEMG Measurements

The bioelectrical activity of the medial (biceps femoris—BF) and lateral (semimembranosus and semitendinosus—SEM) hamstring muscles was recorded according to SENIAM guidelines [16,17]. The skin was cleaned with alcohol, and surface electrodes (Ag/AgCl) (Sorimex, Polska) were attached at a 2 cm center-to-center distance along the direction of the muscle fibers on the lateral and medial hamstring bellies. The signal was registered using the Noraxon G2 TeleMyo 2400 unit (Noraxon Scottsdale, Arizona, USA). The sEMG signal from the evaluated muscles was measured during 60 s of isometric contraction [15]. The measurement was performed in a prone position with the knee flexed to 60° against resistance. Before the signal recording phase for fatigue assessment, the signal was recorded for 5 s at rest and 3 s during maximum contraction. The resting activity was considered as 0% (minimal), and the peak amplitude was considered as 100% (maximal). The threshold (displayed on the screen during signal recording) relative to 50% between min and max was considered the muscle contraction intensity during fatigue assessment.

#### 2.2.2. sEMG Signal Processing

The sEMG data, sampled at a frequency of 1500 Hz, underwent initial preprocessing steps. This involved applying a band-pass 4th order Butterworth filter with a frequency window ranging from 20 to 500 Hz. Subsequently, the 60 s signals were segmented into 0.5 s windows and processed using the Continuous Wavelet Transform (CWT) tool, employing the Daubechies db4 wavelet family with 128 scales. The median frequency (MDF) was extracted from each 0.5 s window for further analysis [18,19].

Six primary parameters were computed to evaluate muscle performance during an isometric task: median *MDF*; slope; intercept; *RMSE*; *R*^2^; and *d_MDF_*. The median *MDF* represents the average MDF across all 0.5 s windows within the 60 s muscle contraction signal. The slope and intercept parameters correspond to the linear regression coefficients derived from the MDFs across the entire acquired EMG signal, set through the minimization of the coefficient of determination for the dataset. The *R*^2^ parameter represents the coefficient of determination, and the Root Mean Square Error (*RMSE*) parameter was utilized to quantify the goodness-of-fit of the linear regression to the MDF values, as per the following equation:

RMSE=∑i=1T(MDFi−MD^Fi)2T,

where RMSE denotes the root mean square error of the dataset; T represents the number of samples in the dataset (for 0.5 s windows within a 60 s EMG signal, T = 120); *MDF_i_* signifies the MDF of the *i*-th window, and MD^Fi denotes the predicted MDF value by linear regression.

The *d_MDF_* parameter (relative change) quantifies the difference between MDF values at the beginning and end of a task. It is computed using the following equation:

$$d_{MDF}=\frac{{MD\overset{¯}{F}}_{start}-{MD\overset{¯}{F}}_{end}}{{MD\overset{¯}{F}}_{start}}\cdot 100\%,$$

where *d_MDF_* is the change between MDF values at the beginning and the end of the task [%]; ${MD\overset{¯}{F}}_{start}$ is the median MDF value for the first three 0.5 s windows from the analyzed signal, and ${MD\overset{¯}{F}}_{end}$ is the median MDF value for last three 0.5 s windows from the analyzed signal.

The three parameters selected for future analysis were *MDF* (Hz), *MDF slope* (Hz), and *d_MDF_* (Hz).

### 2.3. Statistical Analysis

Statistical analysis was performed using STATISTICA 13.0 Pl software. The Shapiro–Wilk test was conducted to assess data normality. The paired *t*-test was used to determine the differences in muscle fatigue variables between groups, the involved and uninvolved limbs, and the BF and SEM muscles. The effect size was calculated using Cohen’s d and interpreted as small (0.2–0.3), medium (0.5), or large (>0.8) [20]. The hamstring muscles ratio was calculated according to the following formula: Ratio = BFMDF/SEM MDF. Differences were considered statistically significant if the level of test probability was lower than the assumed level of significance (*p* < 0.05).

## 3. Results

### 3.1. Difference between Groups

In the group after ACL reconstruction, the BF and SEM muscles demonstrated lower MDF values with a strong effect size, indicating a significant clinical difference (Table 2).

BF muscle: There was a significant difference in MDF values in the BF muscle between the football players after ACL reconstruction and the controls. This difference was observed in both limbs, with higher MDF values in the uninjured group of players. In the operated limb, the MDF value for the BF muscle among the ACL group was significantly lower than in the control. In the non-operated limb, the MDF values were slightly lower among the ACL group compared to controls, but this difference was not significant (Table 2).

SEM muscle: For the SEM muscle, MDF was also higher in both limbs of the uninjured football players compared to those post ACL reconstruction. With regard to the non-operated limb, the MDF value in SEM for the ACL group was significantly lower than the control. In the operated limb, the MDF was also lower, but the difference was not significant compared to the uninjured players (Table 2).

### 3.2. Differences Between Limbs Within Each Group

In neither group were there any significant differences between MDF values in the limbs of the BF muscle. However, in the group after ACL reconstruction, significantly lower MDF values were observed for the SEM muscle of the non-operated limb compared to the operated one. In the control group, the SEM muscle fatigue variables did not significantly differ between the limbs (Table 3).

### 3.3. Differences between BF and SEM Muscles

In football players after ACL reconstruction, in both limbs, significantly greater fatigue was observed in the SEM muscle compared to the BF. The SEM muscle demonstrated significantly higher MDF slope and greater d_MDF_ changes compared to the BF muscle. In the control group, greater fatigue was also observed for the SEM muscle compared to the BF (Table 4).

### 3.4. BF/SEM Ratio

**Figure 1:**
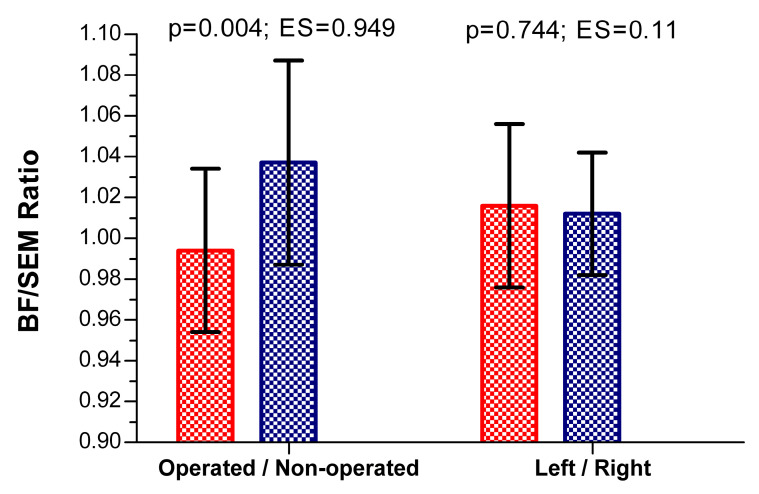
Ratio of MDF between BF and SEM muscles.

In the group after ACL reconstruction, the BF/SEM ratio indicated that in the operated limb, the BF muscle was fatigued to a similar degree as the SEM muscle. However, in the non-operated limb, the SEM muscle demonstrated greater fatigue compared to the BF. A higher BF/SEM ratio in the non-operated limb indicated that the fatigue of the BF was lower in relation to the SEM (Figure 1). In the control group, the BF/SEM ratio was similar in both limbs, indicating only slightly higher MDF in the BF compared to the SEM (Figure 1).

## 4. Discussion

The most important information derived from this study is that football players following ACL reconstruction demonstrate significant asymmetry in fatigue of the lateral and medial hamstring muscles. Another important observation in this study is that football players experience greater fatigue in the SEM compared to the BF muscle, regardless of whether they have undergone ACL reconstruction or not. Moreover, in those after reconstruction, the changes are more pronounced, with higher muscle fatigue in both limbs (they have lower MDF than the non-injured players) and more severe SEM muscle insufficiency (noted in both limbs but with greater intensity in the non-operated one). This study is of clinical relevance because there is a lack of research on how alterations in lateral-to-medial hamstring activation ratio electromyographic activity would be evaluated in football players who were cleared to play post ACL reconstruction. Due to the fact that this method may serve as a rapid and precise diagnostic tool for the evaluation of muscular deficits after ACL reconstruction and because of increased re-injury risk within this muscle group, it has been the subject of analysis and discussion in this work.

Research has indicated that internal tibia rotation may be compromised by harvesting the semitendinosus muscle, which may affect its role as the knee flexor [10]. It has also been indicated that the medial hamstrings were involved in controlling knee rotation, translation, and varus/valgus movements [7,21,22]. It has been reported that when anterior, external rotation, and valgus forces were applied to a knee lacking hamstring support, there was a significant increase in movement in those directions [2,21,23]. Therefore, it has been suggested that the source of overloads leading to ACL injury might be medial hamstring muscle dysfunction and impaired support of the ACL in the transverse and frontal planes [2,21,22].

Many authors have reported that in athletes following ACL reconstruction, muscle strength deficits could be observed many years after reconstruction [8,10], which might impair neuromuscular function and expose the knee joint to an unstable environment during functional tasks [2,24]. It has also been indicated that individuals with a history of ACL reconstruction displayed greater inter-limb differences in hamstring muscle bioelectrical activity compared to healthy controls [5,25]. In our previous study, we noted that lateral-to-medial hamstring asymmetry co-existed with significant strength deficits in both the injured and uninjured limbs compared to footballers post mild injuries and to the control group [8]. In our current study, higher SEM muscle fatigue was observed in the non-operated limb compared to its level following ACL reconstruction, indicating that hamstring muscle asymmetry was visible for a long period after surgery, which could potentially lead to ACL re-injury. This observation may be of significance because this muscle asymmetry could not be subjectively reported by such athletes or be visible in regular orthopedic or physiotherapeutic assessments. The deficits observed in hamstring neuromuscular function years after returning to physical activity suggest the need for targeted continuation of strength training to resolve hamstring impairments upon being cleared to return to activity.

In the study by Guelich et al. [2], the authors emphasized the importance of rotational loading across the knee relating to ACL injury. Although there is little doubt as to the importance of the ACL as a primary restraint to anterior tibia translation [25,26], there is a question as to whether it has significance in controlling axial rotation. The BF is the hamstring muscle most commonly exposed to strain and overuse-related injuries [23,24]. However, the insufficiency of BF performance has also been linked to deficits in the neuromuscular function of the SEM muscle, which may further potentially increase the risk of ACL injury [2,22,26]. Shalhoub et al. [6] examined the different effects of individual muscles on ACL strain induced by independently loading the medial and lateral hamstring muscles. In this study, it was demonstrated that the force ratio between the medial-lateral hamstrings affected tibiofemoral anterior–posterior translation, internal–external rotation, and quadriceps loads. The higher force ratio of the BF compared to SEM led to the greatest tibia external rotation. Conversely, shifting the force ratio from the BF to the SEM caused increased internal rotation of the tibia [6]. Therefore, the greater fatigue of the SEM compared to BF observed in our study may cause a disruption in hamstring performance, leading to excessive BF muscle activity, which, in turn, could potentially result in tibia external rotation and valgus knee [5,6].

It has been stated that hamstring injury could lead to general alterations in sagittal knee joint function, but the medial and lateral hamstrings have different roles in knee control at the coronal plane [2]. The lateral hamstrings flex, rotate externally, and abduct the tibia, reducing ACL strain during knee flexion; however, the medial hamstrings flex, rotate internally, and adduct the tibia [2,9]. Nonetheless, the medial hamstrings contribute much less to strain reduction in the ACL; hence, an appropriate balance between SEM and BF performance is crucial in the ACL graft rupture [2,25]. An important observation of our study is that the football players experienced greater fatigue in the SEM compared to the BF, regardless of whether they had undergone ACL reconstruction or not, and more severe SEM insufficiency was observed in the non-operated limb. The imbalance between the BF and SEM muscles poses a serious threat to the stable functioning of the knee joint. Weakness and excessive fatigue of the SEM cause a situation in which the activity of the BF is no longer counterbalanced by the SEM. In such a scenario, when one muscle starts to work too hard in relation to the other, the directions of forces acting on the ACL also become disrupted. It has been noted that in pivoting sports with fatigued and not well-controlled lateral hamstring muscles, the risk of graft rupture is very high [3,4,27].

Differences in SEM versus BF activation level of the uninjured limb in participants after ACL reconstruction were reported by Briem et al. [10]. During the jump test, the BF muscle demonstrated higher activation levels relative to the SEM, whereas the surgical limb demonstrated similar BF vs. SEM activation levels [10]. The results from our current study support these results because we observed a similar pattern of hamstring insufficiency. However, Briem et al. [10] analyzed which muscle was more active based on sEMG signal amplitude, but we evaluated the sEMG signal frequency, indicating which muscle was more fatigued. Our results showed higher fatigue in the medial hamstring, which probably caused the lateral hamstring to compensate for its insufficiency by being more engaged during the hamstring contraction.

There are no existing studies on the evaluation of the medial and lateral hamstring fatigue asymmetry in football players after ACL reconstruction; thus, our work is the first in which this issue has been examined. This matter seems to be of great significance for rehabilitation and also for the timing of return to sport. Since the knee joint operates in three planes of motion, it is extremely important to ensure full stabilization in all three directions. It was reported that during hamstring exercises, external rotation of the foot changed the ratio of medial to lateral muscle activation to favor the lateral hamstrings, and internal rotation changed the ratio to favor the medial hamstrings [13]. Therefore, selective and differential training may be developed between the medial and lateral hamstring muscles to help better control ACL loading [3,13].

The practical application of this work could be some of the guidelines for the rehabilitation of patients after ACL reconstruction. Based on our results, we may suggest selective strengthening of the medial hamstrings through exercises that incorporate internal foot rotation. This aims to selectively stimulate the SEM muscle in order to restore the balance between SEM and BF. Additionally, since football players, after ACL reconstruction, demonstrated lower MDF in both limbs compared to the control group, indicating general chronic fatigue of the hamstrings, we suggest that such athletes should undergo more frequent recovery and regeneration treatments, which could reduce the risk of graft rupture.

The present study also has some limitations that should be addressed. We have evaluated only male football players; consequently, future research is required that would include female athletes as well as athletes from sports other than football. The limitation of this study is the focus on a specific age group of athletes. The results may not be fully applicable to younger or older athletes or recreational players, as the physiological and biomechanical characteristics can vary across different age groups and levels of play. Furthermore, the study design was observational, and the subjects were assessed only once; thus, longitudinal monitoring of hamstring muscle bioelectrical activity would be of interest. However, by analyzing the biomechanical action of the lateral and medial parts of the hamstring muscle on the dynamic function of the ACL, as well as changes in pathological conditions such as ACL reconstruction, it is possible to infer which part of the muscle undergoes greater overload and is consequently subjected to higher fatigue. Therefore, the higher fatigue of particular hamstring muscles may indicate which direction of knee stabilization by the ACL is at risk of overload. A muscle that becomes fatigued and inefficient more quickly also becomes ineffective in performing its function sooner, which can lead to increased overloading forces acting on the ACL graft. The higher SEM muscle fatigue observed in this study influenced the lateral-to-medial activation ratio within the hamstring muscle, which may probably cause insufficiency of this muscle in laterally stabilizing the knee in the frontal and transverse planes. Thus, it may be suggested that such alterations in fatigue symmetry between the lateral and medial hamstrings could be considered as factors leading to increased risk of primary ACL as well as graft rupture. We suggest that this asymmetry observed in the fatigue ratio is a good indicator for assessing hamstring dysfunction, which can be a valuable guideline for therapy to prevent ACL re-injury. Moreover, it was observed that both limbs in the group post ACL reconstruction had lower MDF compared to football players without injuries. Therefore, we can assume that the hamstring muscles following reconstruction are weaker and more fatigued in both limbs and possibly even worse in the non-operated limb, which likely compensates for the inefficiency of the operated one. Such bilateral weakness of the hamstring muscles may be another risk factor for ACL graft rupture.

## 5. Conclusions

The higher SEM muscle fatigue observed in this study influenced the lateral-to-medial activation ratio within the hamstring muscle, which may be a probable cause of this muscle’s insufficiency in laterally stabilizing the knee in the frontal and transverse plane. Furthermore, the hamstring muscles after reconstruction were more fatigued in both limbs, which may be another risk factor for ACL graft rupture. Therefore, increased fatigue in specific hamstring muscles may indicate the direction in which knee stabilization is compromised due to ACL overload. A muscle that becomes fatigued and inefficient more quickly also becomes ineffective in performing its function sooner, which can lead to increased overloading forces acting on the ACL graft. The symmetry of lateral and medial hamstring muscle fatigue may be proposed as a quick and sensitive method for hamstring muscle imbalance detection, which may be useful in the assessment of re-injury risk following ACL reconstruction.

## Figures and Tables

**Table 1:** Subjects’ characteristics.

	Group 1	Group 2
Number of subjects (n)	25	26
Height (cm)	173 ± 6	178 ± 4
Weight (kg)	75.2 ± 5.6	77.4 ± 6.2
Age	22.9 ± 4.7	23.1 ± 3.2

**Table 2:** Comparison of muscle fatigue variables between groups.

Outcome Measure	BF				SEM			
	Group 1	Group 2	*p*	ES	Group 1	Group 2	*p*	ES
O/L MDF (Hz)	80.14 ± 4.35	84.30 ± 5.08	**0.005**	**0.87**	80.72 ± 5.12	83.02 ± 3.66	0.09	0.51
O/L Slope (Hz)	−0.06 ± 0.05	−0.07 ± 0.04	0.39	0.22	−0.09 ± 0.03	−0.11 ± 0.05	0.13	0.48
O/L d_MDF_ (Hz)	8.38 ± 5.79	9.03 ± 7.51	0.74	0.09	12.62 ± 7.99	14.44 ± 9.13	0.47	0.21
NO/R MDF (Hz)	80.67 ± 3.58	83.01 ± 6.41	0.13	0.45	77.96 ± 5.23	82.06 ± 6.23	**0.02**	**0.71**
NO/R Slope (Hz)	−0.06 ± 0.03	−0.07 ± 0.05	0.68	0.24	−0.09 ± 0.05	−0.12 ± 0.07	0.20	0.49
NO/R d_MDF_ (Hz)	9.63 ± 8.32	9.02 ± 8.07	0.80	0.07	15.79 ± 8.39	10.67 ± 10.32	0.07	0.54

Values are expressed as mean ± SD; *p*—*p*-value; ES—effect size; O—operated leg, NO—non-operated leg; L—left leg; R—right leg; BF—biceps femoris; SEM—semimembranosus and semitendinosus; MDF—median frequency; d_MDF_—median frequency difference.

**Table 3:** Comparison of muscle fatigue variables between limbs within each group.

Outcome Measure	Group 1				Group 2			
	O	NO	*p*	ES	L	R	*p*	ES
BF MDF (Hz)	80.14 ± 4.35	80.67 ± 3.58	0.57	0.13	84.30 ± 5.08	83.01 ± 6.41	0.13	0.22
BF Slope (Hz)	−0.06 ± 0.05	−0.06 ± 0.03	0.61	0.00	−0.07 ± 0.04	−0.07 ± 0.05	0.91	0.00
BF d_MDF_ (Hz)	8.38 ± 5.79	9.63 ± 8.32	0.47	0.17	9.03 ± 7.51	9.02 ± 8.07	0.99	0.00
SEM MDF (Hz)	80.72 ± 5.12	77.96 ± 5.23	**0.04**	**0.53**	83.02 ± 3.66	82.06 ± 6.23	0.25	0.18
SEM Slope (Hz)	−0.09 ± 0.03	−0.09 ± 0.05	0.59	0.00	−0.11 ± 0.05	−0.12 ± 0.07	0.65	0.16
SEM d_MDF_(Hz)	12.62 ± 7.99	15.79 ± 8.39	0.15	0.38	14.44 ± 9.13	10.67 ± 10.32	0.09	0.38

Values are expressed as mean ± SD; *p*—*p*-value; ES—effect size; O—operated leg; NO—non-operated leg; L—left leg; R—right leg, BF—biceps femoris; SEM—semimembranosus and semitendinosus; MDF—median frequency; d_MDF_—median frequency difference.

**Table 4:** Comparison of muscle fatigue variables between BF and SEM muscles.

Outcome Measure	Group 1				Group 2			
	BF	SEM	*p*	ES	BF	SEM	*p*	ES
O/L MDF (Hz)	80.14 ± 4.35	80.72 ± 5.12	0.47	0.12	84.30 ± 5.08	83.02 ± 3.66	0.16	0.28
O/L Slope (Hz)	−0.06 ± 0.05	−0.09 ± 0.03	**0.0002**	**0.72**	−0.07 ± 0.04	−0.11 ± 0.05	**0.0001**	**0.88**
O/L d_MDF_ (Hz)	8.38 ± 5.79	12.62 ± 7.99	**0.009**	**0.60**	9.03 ± 7.51	14.44 ± 9.13	**0.007**	**0.64**
NO/R MDF (Hz)	80.67 ± 3.58	77.96 ± 5.23	**0.003**	**0.60**	83.01 ± 6.41	82.06 ± 6.23	0.20	0.15
NO/R Slope (Hz)	−0.06 ± 0.03	−0.09 ± 0.05	**0.005**	**0.72**	−0.07 ± 0.05	−0.12 ± 0.07	**0.005**	**0.82**
NO/R d_MDF_ (Hz)	9.63 ± 8.32	15.79 ± 8.39	**0.001**	**0.73**	9.02 ± 8.07	10.67 ± 10.3	0.38	0.17

Values are expressed as mean ± SD; *p*—*p*-value; ES—effect size; O—operated leg; NO—non-operated leg; L—left leg; R—right leg; BF—biceps femoris; SEM—semimembranosus and semitendinosus; MDF—median frequency; d_MDF_—median frequency difference.

## Data Availability

All data generated or analyzed during this study are included in this published article.
